# Allogeneic mesenchymal stem cells improve the wound healing process of sheep skin

**DOI:** 10.1186/s12917-018-1527-8

**Published:** 2018-06-25

**Authors:** T. Martinello, C. Gomiero, A. Perazzi, I. Iacopetti, F. Gemignani, G. M. DeBenedictis, S. Ferro, M. Zuin, E. Martines, P. Brun, L. Maccatrozzo, K. Chiers, J. H. Spaas, M. Patruno

**Affiliations:** 1 Department of Comparative Biomedicine and Food Science, University of Padua, Viale dell’Università 16, 35020, Legnaro – Agripolis, Padua, Italy; 2 Department of Animal Medicine, Production and Health, University of Padua, Padua, Italy; 3 Consorzio RFX, Padua, Italy; 4 Department of Molecular Medicine, University of Padua, Padua, Italy; 5 Department of Pathology, Bacteriology and Poultry Diseases, University of Gent, Ghent, Belgium; 6 Global Stem cell Technology-ANACURA group, Noorwegenstraat 4, 9940 Evergem, Belgium

**Keywords:** Wound healing, Mesenchymal stem cells, Cell therapy, Regenerative medicine

## Abstract

**Background:**

Skin wound healing includes a system of biological processes, collectively restoring the integrity of the skin after injury. Healing by second intention refers to repair of large and deep wounds where the tissue edges cannot be approximated and substantial scarring is often observed. The objective of this study was to evaluate the effects of mesenchymal stem cells (MSCs) in second intention healing using a surgical wound model in sheep. MSCs are known to contribute to the inflammatory, proliferative, and remodeling phases of the skin regeneration process in rodent models, but data are lacking for large animal models. This study used three different approaches (clinical, histopathological, and molecular analysis) to assess the putative action of allogeneic MSCs at 15 and 42 days after lesion creation.

**Results:**

At 15 days post-lesion, the wounds treated with MSCs showed a higher degree of wound closure, a higher percentage of re-epithelialization, proliferation, neovascularization and increased contraction in comparison to a control group. At 42 days, the wounds treated with MSCs had more mature and denser cutaneous adnexa compared to the control group. The MSCs-treated group showed an absence of inflammation and expression of CD3+ and CD20+. Moreover, the mRNA expression of hair-keratine (hKER) was observed in the MSCs-treated group 15 days after wound creation and had increased significantly by 42 days post-wound creation. Collagen1 gene (Col1α1) expression was also greater in the MSCs-treated group compared to the control group at both days 15 and 42.

**Conclusion:**

Peripheral blood-derived MSCs may improve the quality of wound healing both for superficial injuries and deep lesions. MSCs did not induce an inflammatory response and accelerated the appearance of granulation tissue, neovascularization, structural proteins, and skin adnexa.

## Background

Skin is a multilayer organ that primarily functions as a protective barrier against the external environment, preventing dehydration and the penetration of external microorganisms [1]. Loss of the integrity of large portions of the skin, as a result of injury, may result in health issues, and poor quality of life [2]. Wound healing is a complex process that begins after injury and proceeds through three phases: hemostasis and inflammation, proliferation, and remodeling [3–5]. These phases are regulated by various cells, cytokines, and growth factors regulate these phases [3–5].

Wound healing re-establishes the skin’s tensile strength and natural barrier function [6, 7]. Dysfunctional healing can lead to lifelong disability and an economic impact on breeding [8, 9]. To optimize wound healing, cell therapy may be an option for treating extensive and chronic wounds. The presence of mesenchymal stem cells (MSCs) in normal skin [10, 11] and their role in natural wound healing [11, 12] indicates that the use of exogenous MSCs might be a means to treat wounds. MSCs are self-renewing, expandable, and able to differentiate into different cell lineages such as osteoblasts, adipocytes, chondrocytes, tenocytes, and myocytes [13–15]. Although bone marrow is one of the most common sources used to obtain MSCs [16, 17], other less invasive sources were used, such as peripheral blood, adipose tissue, and skin [11, 13, 14, 18–22].

The involvement of MSCs in the wound-healing process is significant. MSCs may regulate and improve the three phases of wound healing [23], contribute to the reduction of inflammation [7, 24], promote angiogenesis, reduce excessive wound contraction, attenuate scar formation [7, 25], and stimulate cell movement during epithelial remodeling [8]. Moreover, the immunosuppressive properties of MSCs allow for their potential use in allogeneic therapy. Although stem cell involvement in cutaneous wound healing has been studied in rodent models [22, 25, 26], this process has not been evaluated extensively in large animal models.

The aim of this study was to evaluate the specific effects of allogeneic MSCs in a sheep surgical wound model based on clinical, histopathological and molecular analyses. Moreover, macroscopic and microscopic study were carried out for testing the improvement of the regenerate tissue in the presence of MSCs, in the context of natural regeneration.

## Methods

### Animal model

Six female Bergamasca sheep of similar size and age, provided by a local farm, were acclimated to a stall (MAPS Department, University of Padua, Legnaro, Italy) for 2 weeks. Parasitological and biochemistry examinations were performed. The experiment was approved by The Body for the Protection of Animals (OPBA), ministerial decree n° 51/2015-PR released by the Health Department of Italy. Sheep were chosen because they are less neurologically developed than carnivores and equines and have sufficient superficial space on their backs for creation of experimental lesions. Moreover, sheep are also considered a possible research animal model for human medicine too [27–29]. The number of sheep was chosen based on sample size calculation and the “3Rs” principle (replacement-reduction-refinement) [30]. At the end of project, the animals were not sacrificed but located in a teaching farm.

### Isolation of peripheral blood derived MSCs (PB-MSCs) from sheep

MSCs were isolated from the peripheral blood (PB) of six sheep that were not part of the wound model experimental design. From each animal, 100 ml of blood was taken from the jugular vein using a vacutainer containing the anticoagulant Li-heparin. The mononuclear cells were isolated using the protocol of Martinello et al. [13]. Briefly, the blood was diluted 1:1 with PBS (phosphate-buffered saline) and placed on Ficoll-paque solution (Amersham Biosciences) to obtain mononuclear cells in interphase after centrifugation. Cultures were maintained at 37 °C with 5% CO_2_ in growth medium (DMEM 5671, Sigma-Aldrich) with 10% FCS (fetal bovine serum, Euroclone). On the day of application, PB-MSCs were trypsinized with 0.25% trypsin-EDTA (Euroclone, Italy) and resuspended in hyaluronic acid (Hyalgan®, Fidia).

### Experimental design

In respect of the 3Rs principle [30], six lesions were performed according to the protocol established by Broeckx et al. [31], equidistant and symmetrical with each other on the back of six sheep to analyze the effect of five different therapeutic treatments (three conventional topical cream or gel, cold ionized plasma and MSCs). The distance of each lesion did not influence the result of trials. Six full-thickness square wounds (4 × 4 cm) were created on the back of six sheep. The lesions were created using a scalpel and a square guide model under sterilize surgical condition while the animals were under general anesthesia with analgesia [31]. In the present study, only the PB-MSCs treatment was evaluated and compared to phosphate saline buffer (PBS), used as placebo treatment.

In all six sheep, 1 × 10^6^ PB-MSCs diluted in 1 ml of PBS were injected in the margins of the lesion dedicated to the MSCs study, and 1 × 10^6^ PB-MSCs diluted in 1 ml of hyaluronic acid were topically applied at the center of the same lesion. In the control lesions of all six sheep, PBS only was administered topically to the wounds. After the application of the treatments, the lesions were bandaged with sterile gauze using the “wet-to-dry” method. The wounds were cleaned daily with PBS and the bandages were changed daily.

At two different time points (15 and 42 days from the induction of the lesions), two samples for each lesion treated with PB-MSCs and two samples for each control lesion were collected by means of a 6-mm punch biopsy with appropriate sedation and analgesic drug administration. Of the two collected samples for each time point, one was used for histopathological and immunohistochemistry protocols and one for molecular analyses.

### Clinical evaluation

Lesion appearance was documented daily with photographs, using a ruler to measure wound size. Every week, the same-blinded investigator performed a clinical evaluation of the study animals. The observations were catalogued using the scoring system developed by Hadley et al. [32] (Table 1). The percentages of re-epithelialization and wound contraction were measured at 7, 14, 21, 28 and 42 days post-wound creation.

**Table 1:** Skin-healing parameters scored in the experiment

Parameter	Score
Presence of exudate	1 absent
	2 small
	3 moderate
	4 abundant
Color of exudate	1 clear
	2 pink/red
	3 brown
	4 yellow
	5 green
Character of exudate	1 serous
	2 serosanguineous
	3 sanguineous
	4 purulent +
	5 purulent ++
	6 purulent +++
Gauze	1 dry/clean
	2 dry/stained
	3 moist
	4 wet
Hydration	1 Normal
	2 Maceration +
	3 Maceration ++
	4 Desiccation +
	5 Desiccation ++

### Histopathological analysis

All 24 biopsy samples (6 PB-MSC at day 15, 6 PB-MSC at day 42, 6 control at day 15, 6 control at day 42) were used for histological evaluation and were glowed in OCT (Kaltek) and frozen in isopentane and liquid nitrogen. Samples were cut with cryostat into 5 μm slices before being mounted on slides and stained with Hematoxylin and Eosin (H&E). In order to obtain a full thickness examination, all samples were examined at difference depth (six chosen points). The presence of dermal and subcutaneous infiltrates, (immature) granulation tissue, undifferentiated mesenchymal tissue, and the development of adnexa were evaluated and scored using a 0 to 4 scale (0 absence, 1 presence, 2 small amount, 3 moderate amount, 4 abundant amount). Data were presented as percentage of relative frequency of the assigned values and calculated for each subject and for each parameter.

### Immunohistological evaluation

The serial slices used for histopathological analysis were immunostained with polyclonal rabbit anti-human CD3 (Dako, 1:100), polyclonal rabbit anti-human CD20 (Thermo Fisher, 1:100), monoclonal mouse anti-human MHCII (Dako, 1:40), monoclonal mouse anti-human Ki67 (Dako, 1:10), and monoclonal rabbit anti-human vWF (Dako; 1:3200) antibodies. Immunolabeling was achieved with a high-sensitive horseradish spell out (PO) mouse or rabbit diaminobenzidine kit, with blocking of endogenous PO (Envision DAB+kit; Dako) in an autoimmunostainer (Cytomation S/N S38–7410-01; Dako). An antibody diluent (Dako), with background-reducing components was used to block hydrophobic interactions. The average of three fields from each slice was used to quantitatively evaluate different immunohistological parameters and all measurements were performed with a computer-based program (Leica microscope DM LB2 with Leica Application Suite LAS V4.0) using 20× magnification.

### Real-time PCR analysis of Col1α1 and hKER gene expression

All 24 biopsy samples (6 PB-MSC at day 15; 6 PB-MSC at day 42; 6 control at day 15; 6 control at day 42) were used for molecular biology. Total RNA extraction was performed using Trizol (Life Technologies) reagent and quantified on a Nanodrop spectrophotometer (Thermo Scientific). The complementary DNA (cDNA) was synthetized to perform Real-Time PCR using the ABI 7500 Real-Time PCR system (Applied Biosystems) to evaluate Collagen 1α1 (Col1α1) and hair keratin (hKER) gene expression. All samples were tested in triplicate and untreated skin was used as a calibrator sample. The 2-ΔΔct method was used to analyze and normalize the RNA expression of the target genes with respect to endogenous housekeeping genes.

RPS24 - forward 5’ TTTGCCAGCACCAACGTTG 3′,

reverse 5’ AAGGAACGCAAGAACAGAATGAA 3′,

18S - forward 5’ AAACGGCTACCACATCCAAG 3′,

reverse 5’ TCCTGTATTGTTATTTTTCGTCAC 3′.

PCR primers were designed using Primer Express 3.0 software (Applied Biosystems). The sequences for the forward and reverse primers specific for each mRNA were as follows:

COL1α1 – forward 5’ GTACCATGACCGAGACGTGT 3′,

reverse 5’ AGATCACGTCATCGCACAGCA 3′;

hKER – forward 5’ TGGTTCTGTGAGGGCTCCTT 3′,

reverse 5’ GGCGCACCTTCTCCAGGTA 3′.

### Statistical analysis

Data on clinical, histological, molecular, and immunohistochemical parameters were analysed using PROC MIXED, with animal as a random effect and repeated effect. The statistical linear model included the fixed effect of treatment (MSCs vs Placebo), time (week1, 2, 3, 4, 5, 6) and their interaction. The assumptions of the linear model were graphically inspected using residuals plots. For data that were not normally distributed (Shapiro-Wilks test < 0.90), the Mann-Whitney test was used (wound closure time, % of re-epithelialization and contraction, presence of exudate). The level of statistical significance was set at *p* < 0.05.

## Results

### Assessment of the healing process

**Fig. 1:**
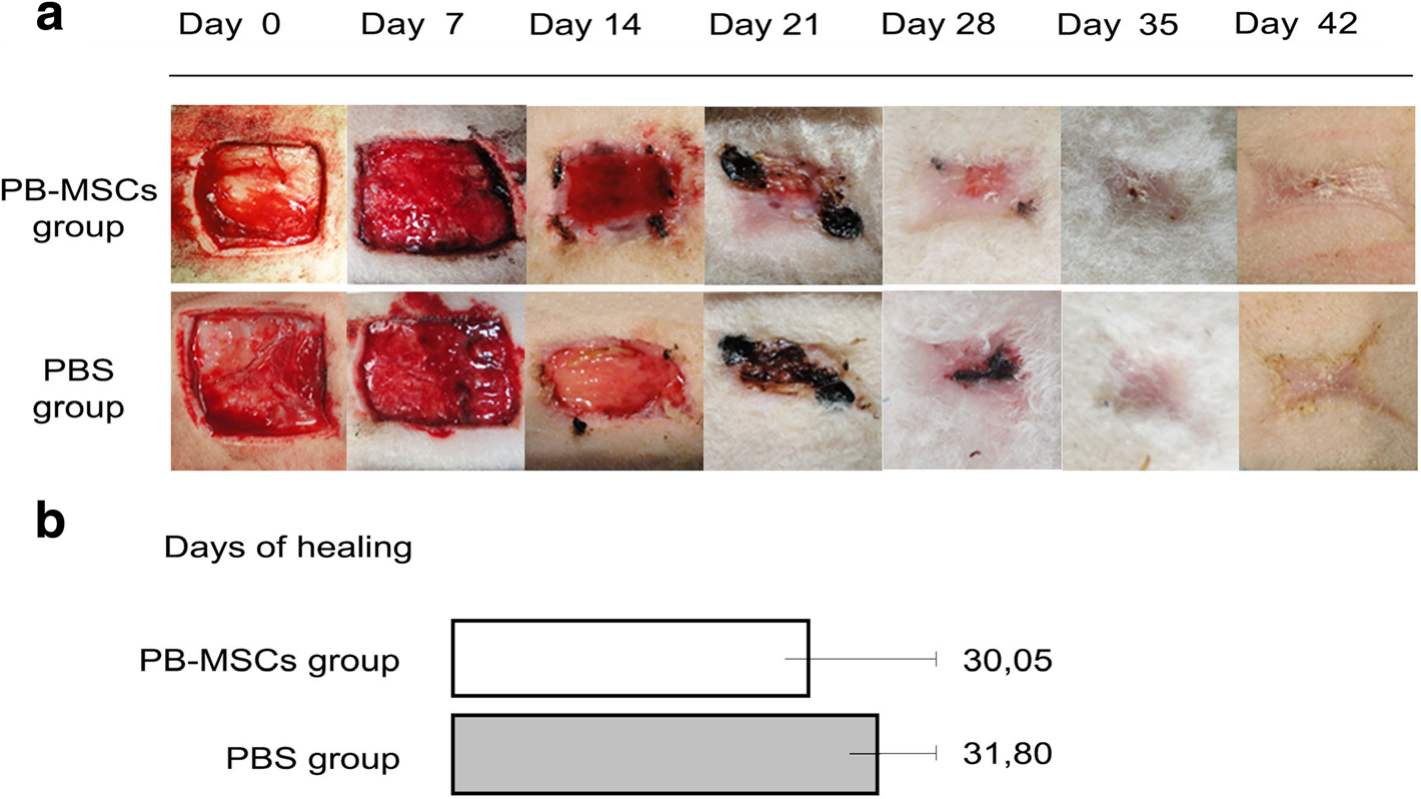
Macroscopic analysis and the percentage of days of healing. **a** Serial macroscopic images of the wound site at different time points after PB-MSCs and PBS treatment. Between day 21 and 28, a smaller wound diameter and higher wound closure rate was observed in PB-MSCs-treated wounds. **b** The panel represents the percentage of days of healing. The wound closure time of the PB-MSC treated wounds (30,05 days) was slightly faster respect than the PBS-treated group (31,80 days)

**Fig. 2:**
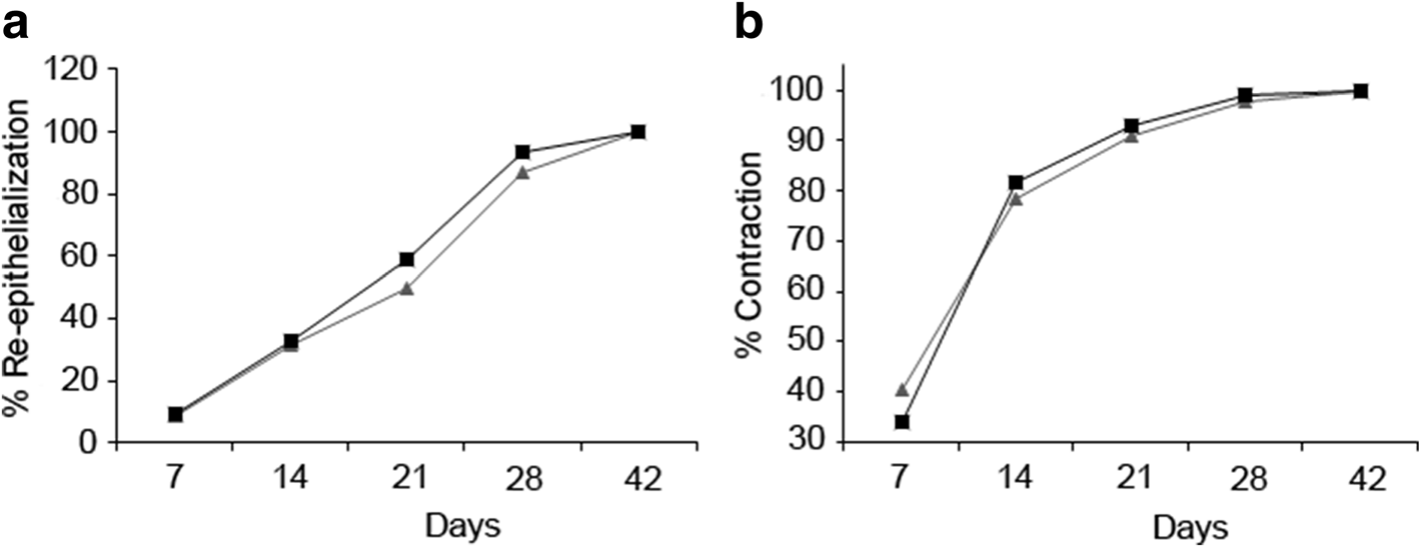
Re-epithelialization and skin contraction. **a** The percentage of re-epithelization. **b** Percentage of contraction after 7, 14, 21, 28 and 42 days of treatment. PB-MSCs-treated wounds trend is represented by black lane, while PBS control group is indicated in grey lane

Wound closure time for the PB-MSCs treated wounds was slightly quicker than that of the control group, average of wound closure time of six sheep was respectively 30.05 and 31.80 days (Fig. 1a-b). However, this was not a significant difference. Two weeks after wound creation, all animals in both the PB-MSCs-treated group and the control group had less than 40% re-epithelialization. Between day 14 and 28, the PB-MSCs-treated lesions had a higher percentage of re-epithelialization in comparison with the control group (58.69% vs 49.89% at day 21 and 93.5% vs 87% at day 28). However, this was not a significant difference. After 42 days of treatment, all wounds had 100% re-epithelialization(Fig. 2a). After two weeks of treatment, the PB-MSCs-treated wounds showed 81% contraction compared to 78% for the control PBS group. However, this was not a significant difference. All lesions had 100% contraction after 42 days of treatment (Fig. 2b).

### Evaluation of skin-healing parameters

The PB-MSCs-treated wounds had a slight, but not-significant increase, in exudate compared to the control group. By the second week, exudate was absent from all lesions in both groups. For all lesions, the color of the exudate was pink/red and changed from serosanguinous to sanguineous over the course of the study.

During the first week post-wound creation, the gauze from all PB-MSCs-treated wounds was dry and clean while those of the PBS control group were slightly moist. However, this was not a significant difference. The wounds, from both groups, showed a normal state of hydration.

### Histopathological examination

Dermal inflammation: at day 15, 33% of PB-MSCs-treated wounds presented with a moderate amount of dermal inflammation, while 67% presented with a small amount. In comparison, after 15 days, 50% of the control group presented with a moderate amount of dermal inflammation and 50% presented with a small amount. After 42 days, inflammation was completely absent in the PB-MSCs treated group, while 60% of the control group presented with a small amount of inflammation.

Subcutaneous inflammation: at day 15, 83% of PB-MSCs-treated wounds contained a small amount of subcutaneous inflammation. In contrast, 17% of the control group presented with moderate and 67% presented with a small amount of inflammation. After 42 days, subcutaneous inflammation was absent in all samples.

**Fig. 3:**
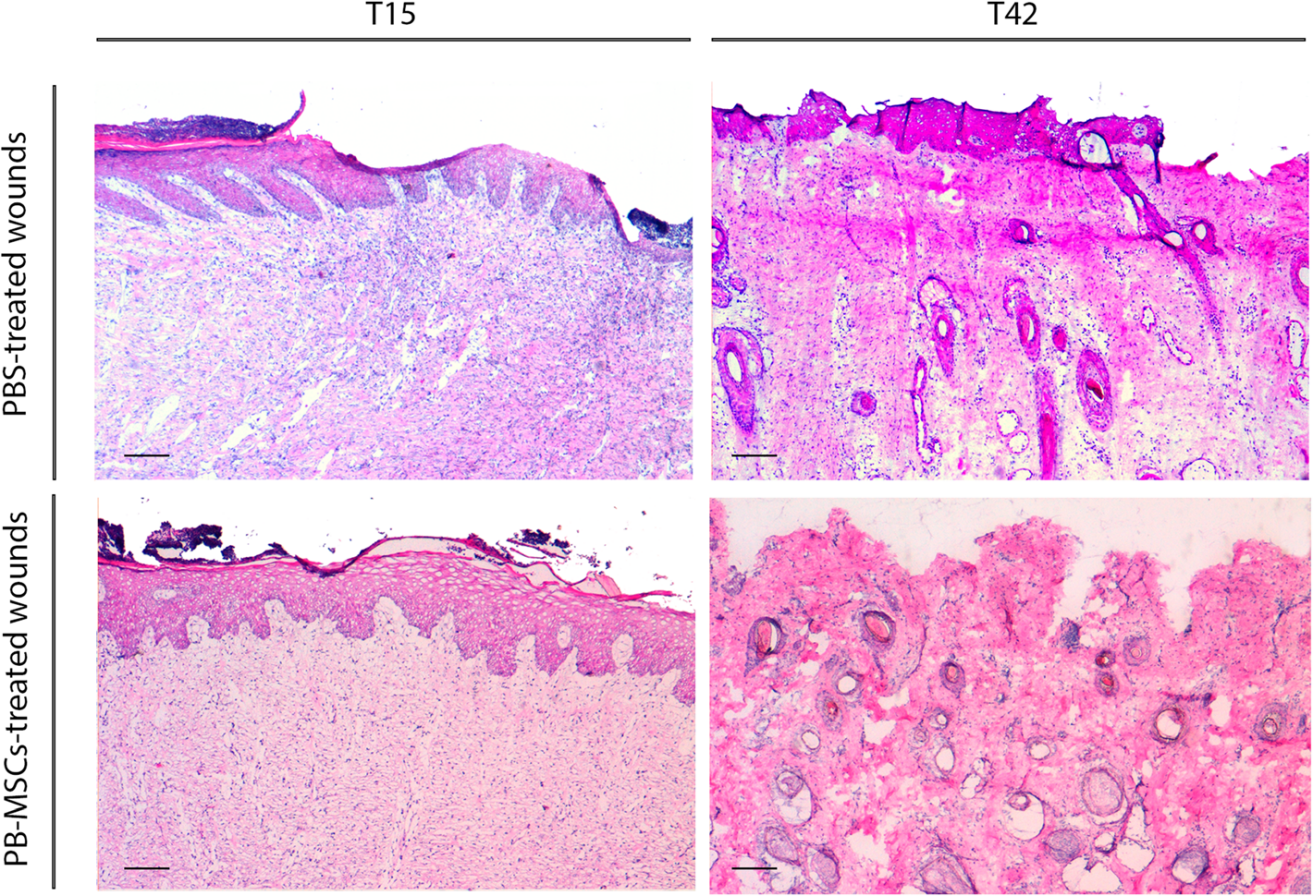
Representative photomicrographs of PBS and PB-MSCs treated wounds (Hematoxylin-Eosin). Photomicrographs of PBS and PB-MSCs treated wounds analyzed at 15 and 42 days from treatments. The images show the presence of immature granulation tissue at 15 days, while mature connective tissue and developing cutaneous adnexa are present at 42 days. The lack of epidermis in representative image of PB-MSCs treated wounds at 42 days is an artefact. Scale bar 151,7 μm

Immature granulation tissue: at day 15, all of the wounds in both groups presented an abundant amount of immature granulation tissue (Fig. 3). Granulation tissue was absent from all wounds by day 42.

Undifferentiated mesenchymal tissue and cutaneous adnexa: undifferentiated mesenchymal tissue and cutaneous adnexa were observed only in samples collected at day 42. Hair follicles, sebaceous, and apocrine glands were present in all samples but the cutaneous adnexa observed in PB-MSCs-treated wounds appeared more mature and denser compare to the control group (Fig. 3).

After 15 days of treatment, ulceration was still present in all the samples. Complete re-epithelization was detected at day 42 in all samples.

### Quantitative analysis of inflammatory, proliferative, vascular and structural factors

Quantitative immunohistochemical staining showed any increase of CD3+ and DC20+ positive cells was similar in both groups. A higher number of MHCII+ cells (*p* < 0.5) was observed after 15 days in PB-MSCs treated wounds (0.45 ± 0,03) compared to control group wounds (0.25 ± 0.02); this was not the case at day 42.

**Fig. 4:**
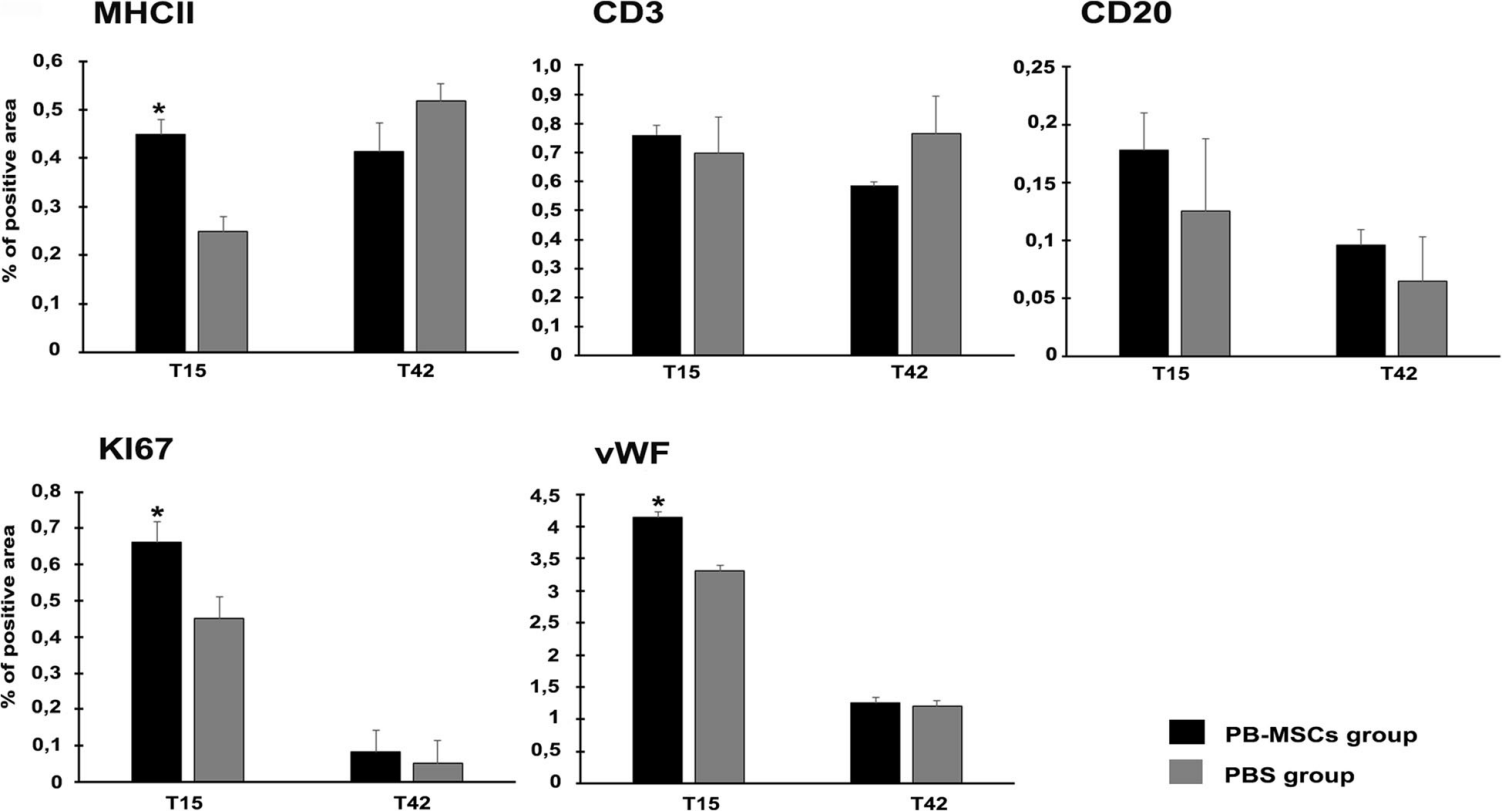
Immunohistochemistry analysis. Percentage of positive staining for CD3, CD20, MHCII, KI67, vWF in PB-MSCs-treated wounds (black bars) and PBS control group (grey bars). Each graph represents the main ± SD of wound treated with PB-MSCs and saline solution PBS. Asterisk indicates significant differences between PB-MSCs group and PBS control group (*p* < 0.05)

Within the newly formed dermis, the lesions treated with PB-MSCs had a higher Ki67 expression (0,661 ± 0,05) compared to the control group (0.313 ± 0,03). After 42 days, Ki67 expression, in both groups, began to decrease (Fig. 4). Using von Willebrand Factor (vWF) antibody staining, more dermal neovascularization was noticed in the PB-MSCs-treated wounds (4.15 ± 0,07) compared with the control lesions (3.32 ± 0,08) (*p* < 0.5). Neovascularization decreased in both groups during the wound healing process, showing the same protein expression values after day 42 (Fig. 4).

**Fig. 5:**
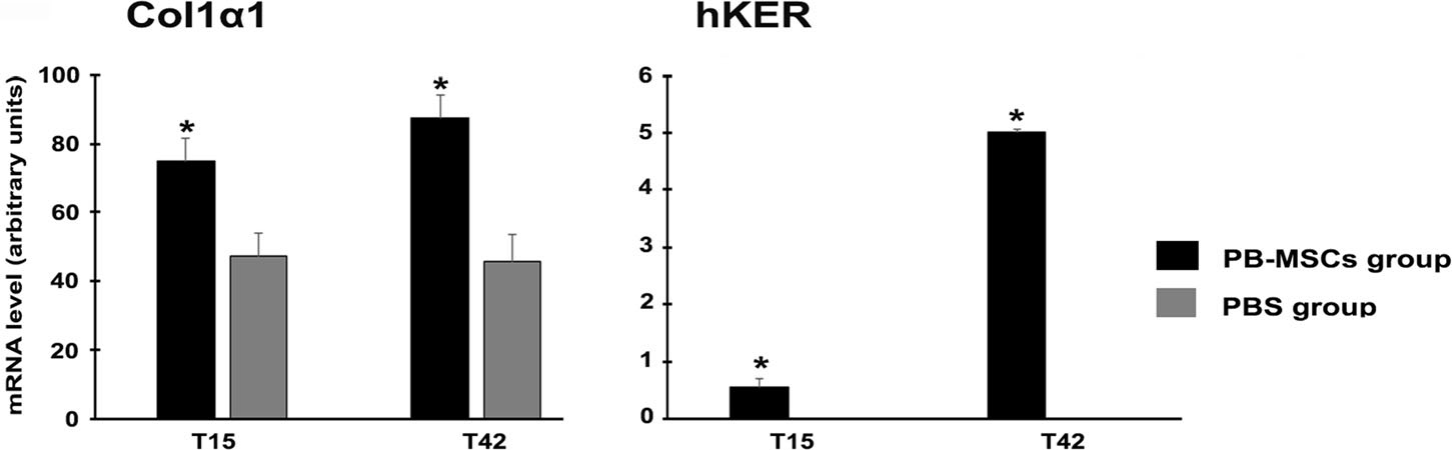
Analyses of mRNA gene expression. mRNA expression of Col1α1 and hKER in PB-MSCs-treated wounds (black bars) and PBS control group (grey bars). Col1α1 and hKER were highly expressed in the treated wounds. Asterisk indicates significant differences between PB-MSCs and PBS control groups (p < 0.05)

The molecular analysis (RT-PCR) of the Col1α1 gene indicated that at day 15 and 42, mRNA expression levels were statistically significant (p < 0.5) in the wounds treated with PB-MSCs (day 15: 75.09 ± 6,5, day 42: 87.65 ± 7,1) compared to the control group (day 15: 47.40 ± 3,6, day 42: 45.80 ± 5,3). PBS treatment did not influence the mRNA expression level of the Col1α1 gene (Fig. 5).

After 15 days, hKER mRNA expression (0.552 ± 0,05) was already present in the wounds treated with PB-MSCs. Furthermore, at day 42, the hKER expression level (5.016 ± 0,1) significantly (p < 0.5) increased in the PB-MSCs-treated lesions, but not in the control group’s lesions. Control PBS alone did not stimulate cutaneous adnexa formation after 15 and 42 days (Fig. 5).

## Discussion

MSCs represent a promising solution to promoting wound healing. The presence of these cells in normal skin [10] suggests their important role in maintaining skin homeostasis. There are different types of stem cells in the epidermis, dermis, and hair follicles [33], which preserve the cellular state of the tissues. Several in vivo studies performed in small laboratory animals have demonstrated that stem cells accelerate wound healing. Many studies have hypothesizing that stem cells contribute to re-epithelization, vascularization, and extracellular remodeling [34–36]. The present study investigated the influence of allogeneic PB-MSCs treatment in a large animal experimental second intention wound healing model, evaluating their short and long-term effects on skin regeneration. Healing associated with a large and/or deep wound in which the tissue edges cannot be approximated is called secondary intention [37]. Wounds are left open to heal with the production of granulation tissue, followed by contraction and epithelialization [38]. Often, this type of healing can be associated with substantial scarring [37]. A previous study, using a murine model, showed that stem cells seeded on a nanostructured membrane helped primary intention healing, such as found with dermal burns [39]. Since MSCs are active in different phases of the healing process, it was hypothesized that they may also be used as a treatment for larger wounds that heal by second intention.

After skin injury, the inflammatory phase starts immediately. During this process, platelets aggregate at the injury site followed by the infiltration of neutrophils, macrophages, and T-lymphocytes [3]. The data presented in this paper show that there was no significant difference in level of inflammation between PBS-treated and PB-MSCs-treated wounds. Microscopic evaluation indicated the presence of the inflammation phase 15 days post-injury in both the PBS control group and the PB-MSCs group at the dermal and subcutaneous levels. A notable result from the study was the complete absence of inflammation after 42 days in the PB-MSCs group whereas 60% of the PBS control group still presented with dermal inflammation. These results corroborate the findings of other studies. For example, Kim et al. [40] showed that experimental full-thickness wounds treated with topical allogeneic MSCs had increased healing and less inflammation, possibly due to the release of immunosuppressive factors in the wound bed that inhibit proliferation of immune cells such as B cells, T cells, and natural killers cells [41, 42]. This effect of allogenic MSCs was shown, in the current study, by absence of an increase of CD3+ and CD20+ cells (B lymphocytes) in MSCs-treated wounds. As discussed by Hussein et al. [43], CD3+ co-receptors helps to activate cytotoxic T lymphocytes, which constitute most of the mononuclear inflammatory cell infiltrate. Moreover, in the last decade, it has been found that MSCs also possess an antimicrobial effect, which helps to reduce excess inflammation from wound contaminants [44] and in the scar formation process [45]. The anti-inflammatory effect of PB-MSCs observed in the current study may result in a shortened inflammatory phase, thereby reducing myofibroblast and fibrocyte development and scar formation [46, 47].

After the inflammation phase, there is the proliferative phase with newly formed granulation tissue that covers the wound area to complete tissue repair. This phase is characterized by angiogenesis, which is important for attracting cytokines, sustaining the granulation tissue, and re-epithelization [48]. Histologically, the granulation tissue, evaluated in this study, was more abundant in wounds treated with PB-MSCs, although the amount of granulation tissue decreased for both cases and controls over time. The newly formed granulation tissue was seen at 15 days post-wound creation both in PBS and PB-MSCs-treated wounds. Evidence of proliferative action by PB-MSCs was confirmed by an increase in Ki67 expression, with this protein present during all active phases of the cell cycle. The PB-MSCs treatment produced a significant increase in Ki67 expression compared to PBS treatment alone, which correlated with the presence of more abundant granulation tissue.

The increase in matrix and vessel formation, after MSCs treatment, may be attributed to the observed up-regulation of growth factors such as EGF, TGF-β1, and stromal-derived growth factor-1α [49]. The more active proliferation induced by PB-MSCs treatment was reflected by an increase in the percentage of re-epithelialization and contraction observed clinically. At 28 days, 93,5% of PB-MSCs-treated wounds were re-epithelized versus 87% of PBS treated wounds. In addition, wound contraction appeared earlier in the PB-MSCs-treated group. The histological data, obtained in this study, confirmed that MSCs might produce multiple pro-angiogenic factors at the lesion site, which stimulate endothelial cells and lead to new blood vessel formation in the wound bed. Revascularization of the wound bed is an important part of the normal wound healing process. Formation of new vessels is necessary to carry blood to the wound area, which requires oxygen and nutrients [50, 51].

The last phase of wound healing is maturation of the tissue. Collagen type 1 is the predominant collagen in normal skin and exceeds collagen type 3 by a ratio of 4:1. During wound healing, this ratio decreases to 2:1 because of an early increase in the deposition of collagen type 3. In this study, the expression of matrix protein collagen 1 was higher in PB-MSCs-treated wounds compared to only treatment with PBS at both 14 and 42 days, indicating an earlier process of wound healing. Moreover, in normal skin, a population of multipotent stem cells capable of generating all of the components of hair, as well as epithelial cells, is located in the hair follicle bulge [52]. These cells do not contribute to preservation of the interfollicular epidermis, but can differentiate into epidermal stem cells after a trauma [53]. In the current study, the treatment of wounds with allogeneic PB-MSCs resulted in the development of new hair follicles and probably also the activation of bulge cells.

Overall, the findings of this large animal study were similar to results from small animal studies. In fact, lesions created in rabbits and dogs [54, 55] demonstrated significantly earlier vascularization, fibroplasia, and maturation of collagen using autologous bone marrow-derived mononuclear cells compared to a control group. Formigli L et al. [56] demonstrated that MSCs seeded on bioengineering scaffolds induced enhanced re-epithelialization characterized by a multilayered epidermis, return of hair follicles, sebaceous glands, and enhanced blood vessel formation. The current study showed that treatment with PB-MSCs leads to a significant increase in the expression of hair keratin mRNA, with expression detectable at 14 days post-wound creation. Furthermore, after 42 days, microscopic evaluation showed an increased in hair follicles, sebaceous and apocrine glands in the PB-MSCs-treated group compared to the control group.

## Conclusion

In the skin regeneration process, PB-MSCs play roles in different phases of wound healing, contributing to the healing process and, as it is confirmed from our paper, does not induce an inflammatory response. Despite some analyzed parameters did not show significant results the trend suggests a beneficial use of PB-MSCs not only for treating superficial injuries, but also for deeper lesions. PB-MSCs were able to speed up the appearance of granulation tissue, stimulate neovascularization, and increase structural proteins and skin adnexa.

## Abbreviations

cDNA: complementary single strand DNA
Col1α1: Collagen 1α1
hKER: hair keratin
IHC: immunohistochemistry
MSCs: mesenchymal stem cells
OPBA: The Body for the Protection of Animals
PB: peripheral blood
PB-MSCs: MSCs isolated from peripheral blood
PBS: phosphate saline buffer
RPS24: ribosomal protein S24
RT-PCR: Real Time-PCR
vWF: von Willebrand factor

## References

[1] Pereira RF, Barrias CC, Granja PL, Bartolo PJ (2013). Advanced biofabrication strategies for skin regeneration and repair. Nanomedicine.

[2] Pereira RF, Bártolo PJ (2016). Traditional therapies for skin wound healing. Advances in Wound Care.

[3] Kondo T (2007). Timing of skin wounds. Legal Med.

[4] McGavin MD, Zachary JF. Pathologic basis of veterinary disease. Fourth edition: Mosby Elsevier; 2007. ISBN-10: 0323028705.

[5] Borena BM (2015). Martens a, Broeckx SY, Meyer E, Chiers K, Duchateau L, Spaas JH. Regenerative skin wound healing in mammals: state-of-the-art on growth factor and stem cell based treatments. Cell Physiol Biochem.

[6] Singer AJ, Thode H, McClain SA (2000). Development of a histomorphologic scale to quantify cutaneous scars after burns. Acad Emerg Med.

[7] Cerqueira MT, Marques AP, Reis LR (2012). Using stem cells in skin regeneration: possibilities and reality. Stem Cells Dev.

[8] Lipinski LC, Wouk AF, da Silva NL, Perotto D, Ollhoff RD (2012). Effects of 3 topical plant extracts on wound healing in beef cattle. Afr J Tradit Complement Altern Med.

[9] Arnold CE, Schaer TP, Baird DL, Martin BB (2003). Conservative management of 17 horses with nonarticular fractures of the tibial tuberosity. Equine Vet J.

[10] Sellheyer K, Krahl D (2010). Cutaneous mesenchymal stem cells. Current status of research and potential clinical applications. Hautarzt.

[11] Maxson S, Lopez EA, Yoo D, Danilkovitch-Miagkova A, Leroux MA (2012). Concise review: role of mesenchymal stem cells in wound repair. Stem Cells Transl Med.

[12] Paquet-Fifield S, Schluter H, Li A, Aitken T, Gangatirkar P, Blashki D, Koelmeyer R, Pouliot N, Palatsides M, Ellis S, Brouard N, Zannettino A, Saunders N, Thompson N, Li J, Kaur P (2009). A role for pericytes as microenvironmental regulators of human skin tissue regeneration. J Clin Invest.

[13] Martinello T, Bronzini I, Maccatrozzo L, Iacopetti I, Sampaolesi M, Mascarello F, Patruno M (2010). Cryopreservation does not affect the stem characteristics of multipotent cells isolated from equine peripheral blood. Tissue Engineering Part C.

[14] Martinello T, Bronzini I, Maccatrozzo L, Mollo A, Sampaolesi M, Mascarello F, Decaminada M, Patruno M (2011). Canine adipose-derived-mesenchymal stem cells do not lose stem features after a long-term cryopreservation. Res Vet Sci.

[15] Gomiero C, Bertolutti G, Martinello T, Van Bruaene N, Broeckx SY, Patruno M, Spaas JH (2016). Tenogenic induction of equine mesenchymal stem cells by means of growth factors and low-level laser technology. Veterinary Research Communication.

[16] Chu CR, Fortier LA, Williams A, Payne KA, McCarrel TM, Bowers ME, Jaramillo D (2018). Minimally manipulated bone marrow concentrate compared with microfracture treatment of full-thickness chondral defects: a one-year study in an equine model. J Bone Joint Surg Am.

[17] Sherman AB, Gilger BC, Berglund AK, Schnabel LV (2017). Effect of bone marrow-derived mesenchymal stem cells and stem cell supernatant on equine corneal wound healing in vitro. Stem Cell Res Ther.

[18] Cheng HY, Ghetu N, Wallace CG, Wei FC, Liao SK. The impact of mesenchymal stem cell source on proliferation, differentiation, immunomodulation and therapeutic efficacy. Journal of Stem Cell Research & Therapy. 2014;4(10)

[19] Lyahyai J, Mediano DR, Ranera B, Sanz A, Remacha AR, Bolea R, Zaragoza P, Rodellar C, Martín-Burriel I (2012). Isolation and characterization of ovine mesenchymal stem cells derived from peripheral blood. BMC Vet Res.

[20] Heidari et al., Comparison of Proliferative and Multilineage Differentiation Potential of Sheep Mesenchymal Stem Cells Derived from Bone Marrow, Liver, and Adipose Tissue. Avicenna J Med Biotechnol Vol. 5, No. 2, April–June 2013. PMC3689554 23799179

[21] Martinello (2013). Effects of in vivo applications of peripheral blood-derived mesenchymal stromal cells (PB-MSCs) and Platlet-rich plasma (PRP) on experimentally injured deep digital flexor tendons of sheep. J Orthop Res.

[22] Hu MS, Rennert RC, McArdle A, Chung MT, Walmsley GG, Longaker MT, Lorenz HP (2014). The role of stem cells during Scarless skin wound healing. Advance in Wound Care (New Rochelle)..

[23] Wu Y, Chen L, Scott PG, Tredget EE (2007). Mesenchymal stem cells enhance wound healing through differentiation and angiogenesis. Stem Cells.

[24] Liu P, Deng Z, Han S, Liu T, Wen N, Lu W, Geng X, Huang S, Jin Y (2008). Tissue-engineered skin containing mesenchymal stem cells improves burn wounds. Artif Organs.

[25] Jackson WM, Nesti LJ, Tuan RS (2012). Concise review: clinical translation of wound healing therapies based on mesenchymal stem cells. Stem Cells Transl Med.

[26] Cerqueira MT, Pirraco RP, Marques AP (2016). Stem cells in skin wound healing: are we there yet?. Advance in Wound Care (New Rochelle).

[27] Music E, Futrega K, Doran MR (2018). Sheep as a model for evaluating mesenchymal stem/stromal cell (MSC)-based chondral defect repair. Osteoarthr Cartil.

[28] Chevrier A, Nelea M, Hurtig MB, Hoemann CD, Buschmann MD (2009). Meniscus structure in human, sheep, and rabbit for animal models of meniscus repair. J Orthop Res.

[29] YR KJ, Evans RG, May CN (2018). An ovine model for studying the pathophysiology of septic acute kidney injury. Methods Mol Biol.

[30] Russel WMD, Burch RL. The principles of Human Experimental Technique UFAW. London: Methuen & Co.; 1959. ISBN: 0900767782 9780900767784.

[31] Broeckx SY, Borena BM, Van Hecke L, Chiers K, Maes S, Guest DJ, Meyer E, Duchateau L, Martens A, Spaas JH (2015). Comparison of autologous versus allogeneic epithelial-like stem cell treatment in an in vivo equine skin wound model. Cytotherapy.

[32] Hadley HS, Stanley BJ, Fritz MC, Hauptman JG, Steficek BA (2013). Effects of a cross-linked hyaluronic acid based gel in the healing of open wounds in dogs. Vet Surg.

[33] Cui P, He X, Pu Y, Zhang W, Zhang P, Li C, Guan W, Li X, Ma Y (2014). Biological characterization and pluripotent identification of sheep dermis-derived mesenchymal stem/progenitor cells. Biomed Res Int.

[34] Yoshikawa T, Mitsuno H, Nonaka I, Sen Y, Kawanishi K, Inada Y, Takakura Y, Okuchi K, Nonomura A (2008). Wound therapy by marrow mesenchymal cell transplantation. Plast Reconstr Surg.

[35] Kwon DS, Gao X, Liu YB, Dulchavsky DS, Danyluk AL, Bansal M, Chopp M, McIntosh K, Arbab AS, Dulchavsky SA, Gautam SC (2008). Treatment with bone marrow derived stromal cells accelerates wound healing in diabetic rats. Int Wound J.

[36] Badillo AT, Redden RA, Zhang L, Doolin EJ, Liechty KW (2007). Treatment of diabetic wounds with fetal murine mesenchymal stromal cells enhances wound closure. Cell Tissue Res.

[37] Iocono JA, Ehrlich HP, Gottrup F, et al. The biology of healing. In: Leaper DL, Harding KG, editors. Wounds: biology and management. Oxford, England: Oxford University Press; 1998. p. 12–22.

[38] You HJ, Han SK (2014). Cell therapy for wound healing. J Korean Med Sci.

[39] Souza CM, Mesquita LA, Souza D, Irioda AC, Francisco JC, Souza CF, Guarita-Souza LC, Sierakowski MR, Carvalho KA (2014). Regeneration of skin tissue promoted by mesenchymal stem cells seeded in nanostructured membrane. Transplant Proc.

[40] Kim JW, Lee JH, Lyoo YS, Jung DI, Park HM (2013). The effects of topical mesenchymal stem cell transplantation in canine experimental cutaneous wounds. Vet Dermatol.

[41] Matthay MA, Goolaerts A, Howard JP, Lee JW (2010). Mesenchymal stem cells for acute lung injury: preclinical evidence. Critical Care Med.

[42] Hass R, Kasper C, Bohm S, Jacobs R (2011). Different populations and sources of human mesenchymal stem cells (MSC): a comparison of adult and neonatal tissue-derived MSC. Cell Communication and Signaling.

[43] Hussein MR, Hassan HI (2006). Analysis of the mononuclear inflammatory cell infiltrate in the normal breast, benign proliferative breast disease, in situ and infiltrating ductal breast carcinomas: preliminary observations. J Clin Pathol.

[44] Mei SH, Haitsma JJ, Dos Santos CC, Deng Y, Lai PF, Slutsky AS, Liles WC, Stewart DJ (2010). Mesenchymal stem cells reduce inflammation while enhancing bacterial clearance and improving survival in sepsis. American Journal of Respiratory Critical Care Medicine.

[45] Nuschke A (2014). Activity of mesenchymal stem cells in therapies for chronic skin wound healing. Organ.

[46] Rhett JM, Ghatnekar GS, Palatinus JA, O’Quinn M, Yost MJ, Gourdie RG (2008). Novel therapies for scar reduction and regenerative healing of skin wounds. Trends Biotechnol.

[47] Jackson WM, Nesti LJ, Tuan RS (2012). Mesenchymal stem cell therapy for attenuation of scar formation during wound healing. Stem Cell Res Ther.

[48] Burnouf T, Goubran HA, Chen T-M, Ou K-L, El- Ekiaby M, Radosevic M (2013). Blood-derived biomaterials and platelet growth factors in regenerative medicine. Blood Rev.

[49] Chen L, Tredget EE, Wu PY, Wu Y (2008). Paracrine factors of mesenchymal stem cells recruit macrophages and endothelial lineage cells and enhance wound healing. PLoS One.

[50] Morimoto N, Yoshimura K, Niimi M (2012). An exploratory clinical trial for combination wound therapy with a novel medical matrix and fibroblast growth factor in patients with chronic skin ulcers: a study protocol. Am J Transl Res.

[51] Zhang Y, Wang T, He J, Dong J (2014). Growth factor therapy in patients with partial-thickness burns: a systematic review and meta-analysis. Int Wound J.

[52] Oshima H, Rochat A, Kedzia C, Kobayashi K, Barrandon Y (2001). Morphogenesis and renewal of hair follicles from adult multipotent stem cells. Cell.

[53] Levy V, Lindon C, Zheng Y, Harfe BD, Morgan BA (2007). Epidermal stem cells arise from the hair follicle after wounding. FASEB J.

[54] Borena BM, Pawde AAM, Aithal HP, Kinjavdekar P, Singh R, Kumar D (2010). Evaluation of autologous bone marrow-derived nucleated cells for healing of full thickness skin wounds in rabbits. Int Wound J.

[55] Borena BM, Pawde Amarpal AM, Aithal HP, Kinjavdekar P, Singh R, Kumar D (2009). Evaluation of healing potential of autologous bone marrow-derived nucleated cells on incisional wounds in dogs. Indian Journal of Veterinary Surgery.

[56] Formigli L, Paternostro F, Tani A, Mirabella C, Quattrini Li A, Nosi D, D'Asta F, Saccardi R, Mazzanti B, Lo Russo G, Zecchi-Orlandini S (2015). MSCs seeded on bioengineered scaffolds improve skin wound healing in rats. Wound Repair Regen.

